# *Lactiplantibacillus plantarum* ameliorated the negative effects of a low-protein diet on growth performance, antioxidant capacity, immune status, and gut microbiota of laying chicks

**DOI:** 10.3389/fmicb.2025.1507752

**Published:** 2025-02-05

**Authors:** Lele Hou, Huiling Qiu, Jihong Dong, Huawei Liu, Shansong Gao, Fu Chen

**Affiliations:** ^1^Institute of Animal Nutritional Metabolic Disease and Poisoning Disease, College of Veterinary Medicine, Qingdao Agricultural University, Qingdao, China; ^2^Institute of Animal Nutritional Metabolic Disease and Poisoning Disease, Haidu College, Qingdao Agricultural University, Laiyang, China; ^3^Institute of Animal Nutritional Metabolic Disease and Poisoning Disease, College of Animal Science and Technology, Qingdao Agricultural University, Qingdao, China

**Keywords:** *Lactiplantibacillus plantarum*, low-protein diet, antioxidant capacity, immune status, gut microflora

## Abstract

This experiment was conducted to investigate the effects of adding *Lactiplantibacillus plantarum* to a low-protein diet on the growth performance, ability immune status, and intestinal microbiota of 0–21-day-old layer chickens. A total of 180 one-day-old healthy Hy-line brown laying chicks were randomly divided into three groups with three replicates each of 20 chicks. The control group was fed a basal diet containing 19% protein, the low-protein (LP) group was fed a diet containing 17% protein, and the probiotic (LPL) group was fed with the 17% protein diet supplemented with *L. plantarum* (1.0 × 10^9^ CFU/kg). The growth performance, antioxidant capacity, immune status, and gut microbiota of laying chickens were detected. We found that *L. plantarum* supplementation increased the activities of superoxide dismutase (SOD) and glutathione peroxidase (GSH-Px), total antioxidant capacity (T-AOC), and levels of immunoglobulin (Ig) A, IgG, and interleukin-10 (IL-10) in serum of 17% protein +1.0 × 10^9^ CFU/kg *L. plantarum* (LPL) compared to the 19% protein group (control). Furthermore, *L. plantarum* supplementation increased the liver index, GSH-Px and T-AOC activity in serum, and changed the microflora structure, diversity, and polyketose unit bioanabolic metabolism of 17% protein +1.0 × 10^9^ CFU/kg L. plantarum (LPL) compared to the 17% protein group (LP). In conclusion, *L. plantarum* supplementation could compensate for the adverse effects of low-protein diets in chicks, and the combination of a low-protein diet and *L. plantarum* is a feasible way to reduce protein in the diet.

## Graphical Abstract

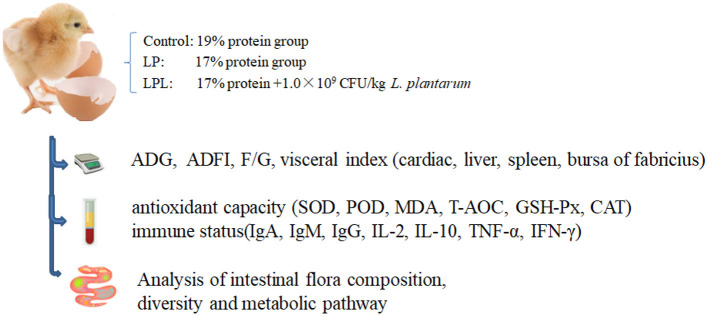

## Introduction

At present, with the rapid development of animal husbandry and the feed industry, protein feed resources are increasingly short, which has become an important factor restricting the further development of animal husbandry (Deck et al., 2023; Cruz et al., 2020). The effective reduction of crude protein in feed is one of the hot topics in livestock raising (Macelline et al., 2021).

Based on the ideal amino acid pattern, the method of reducing the dietary protein level while adding synthetic amino acids has the advantages of improving the utilization rate of livestock and poultry dietary protein, increasing feed conversion rate, reducing feed cost and reducing nitrogen excretion. Wang D. et al. (2023) and Zhu et al. (2022) found the low protein diet supplemented with different kinds of essential amino acids could reduce the fecal nitrogen emission without affecting growth performance and improve the slaughter performance, meat quality of pigs and the structure of gut microbiota. But the low protein diet still has some disadvantages. Wu et al. (2023) found the low protein diet caused the rumen exhibited increased relative abundance of pathogenic microbiota and VFA-degrading microbiota, leading to disruptions in immune homeostasis within the host's ruminal mucosa. Sun et al. (2022) found that low protein diet (14% CP) deficient in Arg (0.80%) result in augmented oxidative damage and impaired development of intestinal mucosa. This situation may be due to some small peptides contained in proteins which can be directly used by passive objects cannot be replaced by the supplement of synthetic amino acids, the utilization rate of a low-protein diet for livestock and poultry is reduced (Macelline et al., 2023). The effective reduction of crude protein in feed is a difficult problem in animal husbandry (Macelline et al., 2021).

Probiotics, including *Lactoplantibacillus plantarum (L. plantarum)*, can produce the digestive enzymes (such as protease, amylase, cellulase, and phytase), which can promote the digestion, absorption and utilization of protein, starch, fiber and fat in the feed (Hosseini et al., 2024; Sampath et al., 2023; Dasriya et al., 2024). Probiotics enter the gastrointestinal tract and colonize the gastrointestinal tract of animals, which can maintain the intestinal microecological balance. The metabolism of probiotics produces large amounts of lactic acid and acetic acid, which can reduce the pH of feed, inhibit harmful pathogens such as *Escherichia coli* and *Salmonella* in feed, and reduce the diarrhea rate of young animals (Li et al., 2023; Du et al., 2023; Juricova et al., 2022). At the same time, the acidification of the animal gastrointestinal tract helps to increase the activity of endogenous protease and thus, improve protein digestibility. Vasquez et al. (2023) found multispecies probiotic supplementation in diet with reduced crude protein levels altered the composition and function of gut microbiome and restored microbiome-derived metabolites in growing pigs. Imari et al. (2023) found adding probiotics to the diet remarkably improved the productive performance and nutrient digestibility of broiler-fed low-protein diets. Therefore, probiotics can be an ideal choice to compensate for the disadvantages of a low-protein diet (Peng et al., 2020). In this study, *L. plantarum* was added to a low-protein diet to observe the changes in growth performance, immune indices, antioxidant capacity and intestinal flora diversity of laying hens, and to evaluate the feasibility of adding *L. plantarum* to a low-protein diet.

## Materials and methods

### Animal ethics statement

All animal experiments were approved by the Qingdao Agricultural University Animal Care and Use Committee (Qingdao, China) in accordance with Laboratory Animal Guidelines for the Ethical Review of Animal Welfare (GB/T35892-2018, National Standards of the People's Republic of China).

### Animals and experimental treatments

A total of 180 one-day-old healthy Hy-line brown laying chicks were randomly divided into three groups with three replicates each of 20 chicks. The control group was fed a basal diet containing 19% protein, the low-protein (LP) group was fed a diet containing 17% protein, and the probiotic (LPL) group was fed with the 17% protein diet supplemented with *L. plantarum* (1.0 × 10^9^ CFU/kg) from laboratory of animal nutritional metabolic disease and poisoning disease. All experimental chicks were raised in ventilated buildings of chicken cage with up and down three floors, each floor three rooms, each room size is 100^*^80^*^50 cm, provided with continuous light, water and feed provided *ad libitum* during the feeding period of 21 days. The formulation and approximate composition of the basal diet and low-protein diet are shown in Table 1.

**Table 1:** Formulation and proximate composition of experimental diets.

	**19% protein diet**	**17% protein diet**
**Ingredient (%)**		
Corn	65.8	69.8
Bran	3.12	4.29
Soybean meal	27.8	22.15
Dicalcium phosphate	1.18	1.2
Stone dust	1.24	1.52
Salt	0.15	0.15
Lysine sulfate	0.08	0.21
DL-methionine	0.13	0.18
Premix^*^	0.5	0.5
Totle	100	100
**Chemical composition (g/kg DM)**		
Metabolic energy (MJ/kg)	11.93	11.92
Crude protein (%)	19.04	17.02
Calcium (%)	0.9	0.9
Available phosphorus (%)	0.4	0.4
Lysine (%)	1	1
Methionine (%)	0.41	0.41
Methionine + cystine (%)	0.73	0.73

* The vitamins and minerals provided (per kg diet): 60.00 mg of iron, 60.60 mg of manganese, 8.00 mg of copper, 80.00 mg of zinc, 0.30 mg of selenium, 0.48 mg of iodine, 8.40 IU of vitamin E, 3.45 IU of vitamin K_3_, 0.8 IU of vitamin B_1_, 3.75 IU of vitamin B_2_, vitamin B_3_ 5.40 mg, vitamin B_5_ 20.00 mg, vitamin B_11_ 0.45 mg, vitamin B_7_ 0.10 mg, vitamin A 8,000 IU, and vitamin D_3_ 2,000 IU.

### Growth performance

On day 21, all chicks were fasted for 12 h and drank water freely. The body weight and feed consumption of chicks in each group were recorded at the beginning and end of the experimental period, and the average daily gain (ADG), average daily feed intake (ADFI), and feed/gain (F/G) of chicks were calculated. After euthanasia, heart, liver, spleen, and bursa of fabricius were separated and weighed quickly. The organ index was calculated according to the formula [Organ index (%) = organ weight (g)/body weight (g)×100%].

### Sample collection and processing

On day 21, six chicks per replicates group were euthanized by cervical dislocation (AVMA Panel on Euthanasia, 2001) and blood was taken from jugular vein and collected in anticoagulant tubes, and serum separated by centrifugation at 3,500 ×g for 10 min (centrifuge3K15, Sigma) and stored at −20°C for analysis of immune factors. The caecum contents from a 2 cm long caecum segment were collected and frozen in liquid nitrogen for analysis of microbiota diversity.

### Determination of antioxidant capacity and immune status in serum

The activities of superoxide dismutase (SOD, A001-3-2), glutathione peroxidase (GSH-Px, A005-1-2), peroxidase (POD, A084-2-1) and catalase (CAT, A007-1-1), malondialdehyde (MDA, A003-1-2) content, and total antioxidant capacity (T-AOC, A015-2-1) in serum were analyzed according to the manufacturer's instructions of the reagent kits (Nanjing Jiancheng Bioengineering Institute, Nanjing, China).

The levels of immunoglobulin (Ig) A (H108-1-2), IgM (H109-1-1), IgG (H106-1-1), interleukin (IL)-2 (H003-1-1), IL-10 (H009-1-2), tumor necrosis factor-α (TNF-α, H052-1-2), and interferon-γ (IFN-γ, H025-1-2) in serum were analyzed according to the manufacturer's instructions of the reagent kits (Shanghai Enzyme-linked Biotechnology Co., Ltd., Shanghai, China).

### DNA isolation and 16S rRNA sequencing

Microbial genomic DNA of all samples from the caecum contents were isolated using the stool DNA kit (Sangon Biotech Co., Ltd., Shanghai, China), the V3–V4 regions of bacterial 16S rRNA were amplified according to specific primers (F:5′-ACTCCTACGGGAGGCAGCA-3′,R:5′-CGGACTACHVGGGTWTCTAAT-3′). PCR reaction conditions was follows: 94°C for 2 min (once only), followed by 30 cycles consisting of 94°C for 30 s, 56°C for 30 s, and 72°C for 30 s. PCR products were sequenced on an Illumina platform (Shanghai Pyseno Biological Co., Ltd., Shanghai, China).

### Processing of 16S rRNA sequencing data

16sRNA data were obtained by double-ended sequencing of community DNA fragments on the Illumina platform. QIIME2 software and the DADA2 method were used to perform priming, quality filtering, denoising, splicing, and chimerization for the original data, which is equivalent to 100% similarity clustering to obtain Amplicon Sequence Variants (ASVs). The obtained ASVs were compared with the Greengenes database using QIIME2 software to obtain the composition of the experimental samples at the phylum and genus levels. QIIME2 software was used to analyze the richness and diversity of the gut microbiota using the Chao1 index and Shannon index of α diversity and weighted UniFrac distance and weighted UniFrac distance of β diversity using the unflattened ASV table. Linear discriminant analysis (LDA) effect size (LEfSe) was used to select the bacterial markers of microbiota among groups with the significantly different represented by LDA score ≥ 2.0. Using PICRUSt2 software and referring to the known microbial genome data, the composition of functional units of the microbiota was predicted for the samples with only the sequencing data of microbiota marker genes, so as to understand the general situation of the functional potential of the microbiota in the tested samples.

### Statistical analysis

The data were presented as the mean ± standard deviation (SD). The data were analyzed with one-way ANOVA using the SPSS 26.0 (Dong et al., 2024). Statistical analyses of microbial composition data were performed using R (R Core Team, 2019). The normality of data distribution was analyzed using the Shapiro–Wilk test. The Kruskal–Wallis test for alpha and beta diversity was performed using the QIIME2 pipeline. Permutational multivariate analysis of variance (PERMANOVA) was used to determine significant differences in the PCoA plot. The significance level was *P* < 0.05, the extremely significant level was *P* < 0.01, the difference was not significant if *P* > 0.05.

## Results

### Growth performance

**Figure 1:**
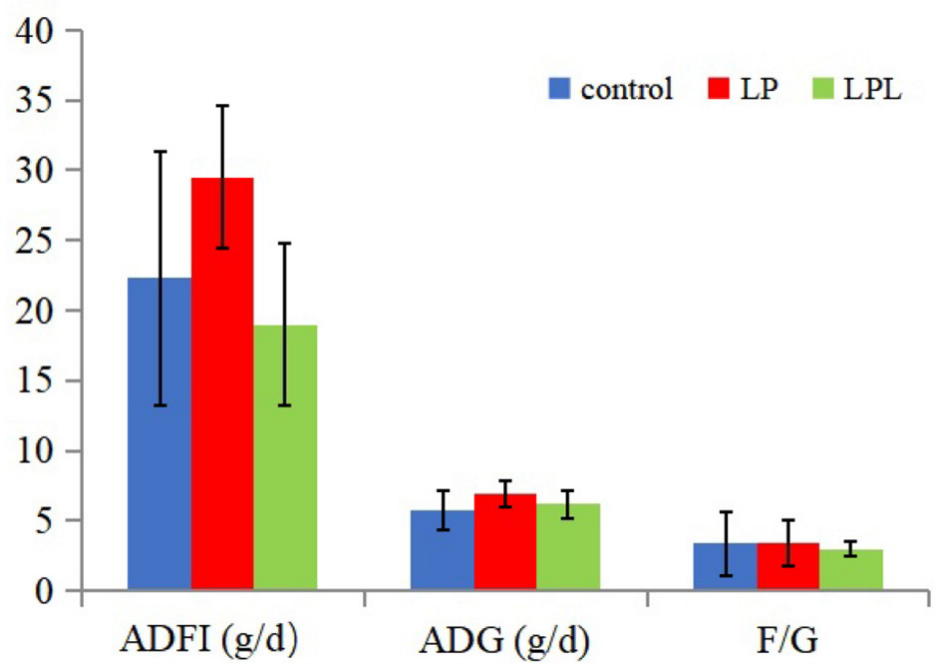
Effect of the low-protein diet containing *L. plantarum* on growth performance of laying chicks. ADFI, average daily feed intake; ADG, average daily weight gain; F/G, ratio of feed to gain; Control group was fed a basal diet containing 19% protein, LP group was fed a diet containing 17% protein, LPL group was fed with the 17% protein diet supplemented with *L. plantarum*; Data are presented as means ± SD (*n* = 6).

**Figure 2:**
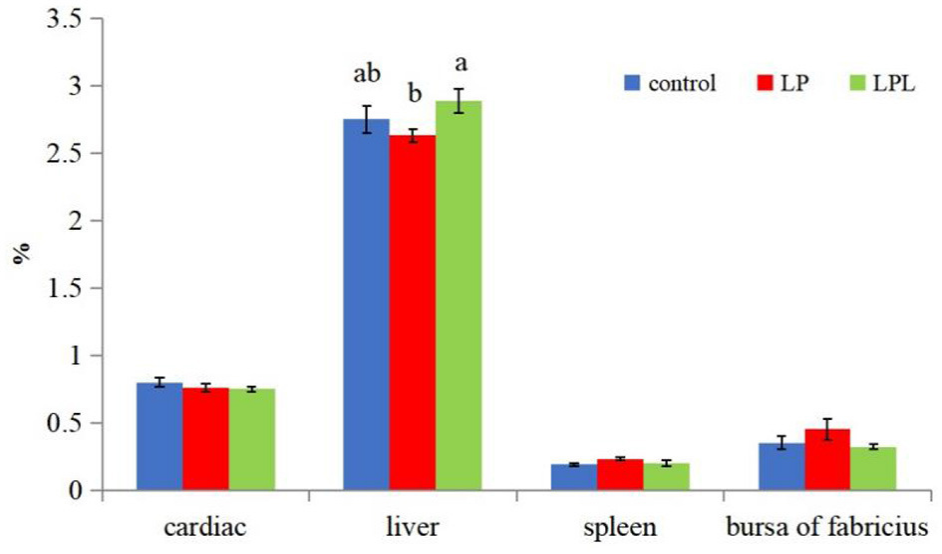
Effect of low-protein diet containing *L. plantarum* on visceral index of laying chicks. Control group was fed a basal diet containing 19% protein, LP group was fed a diet containing 17% protein, LPL group was fed with the 17% protein diet supplemented with *L. plantarum*; Data are presented as means ± SD (*n* = 6); a, b means indicates a significant difference at the *P* < 0.05 level.

The effects of dietary *L. plantarum* supplementation on growth performance of laying chicks are shown in Figure 1. There were no significant differences in ADFI, ADG, and F/G among the three groups (*P* > 0.05). The effects of dietary *L. plantarum* supplementation on the visceral index of laying chicks are shown in Figure 2. Compared with the LP group, the LPL group showed a significantly higher liver index (*P* < 0.05). There was no significant difference in heart, spleen, and bursae of fabricius indices among the three groups (*P* > 0.05).

### Antioxidant capacity

**Figure 3:**
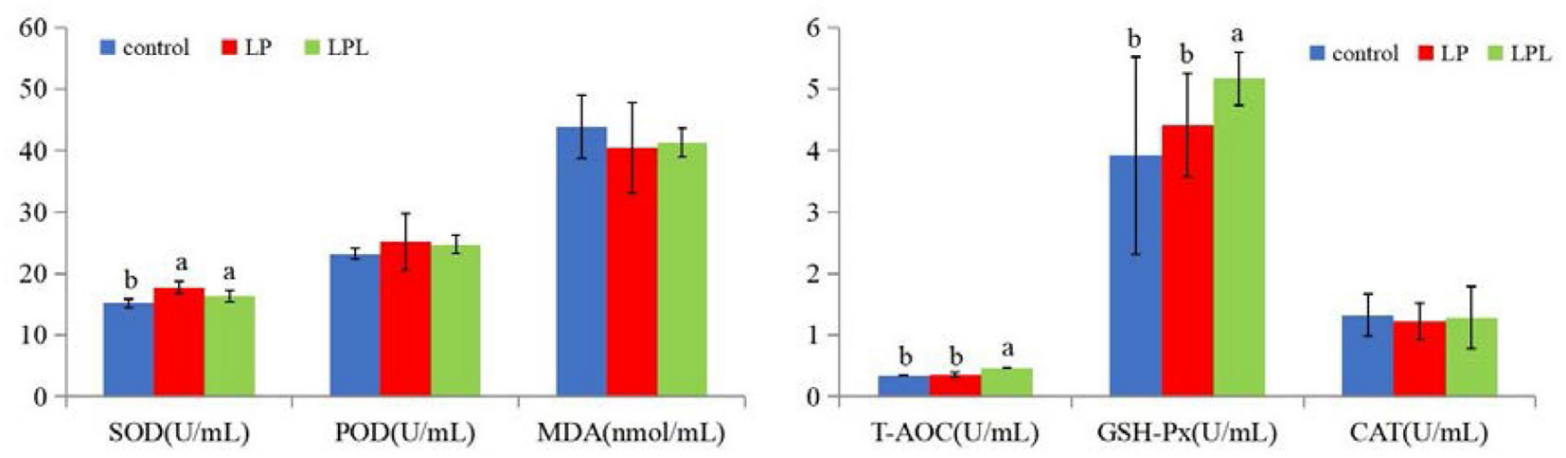
Effect of low-protein diet containing *L. plantarum* on serum antioxidant capacity of laying chicks. Control group was fed a basal diet containing 19% protein, LP group was fed a diet containing 17% protein, LPL group was fed with the 17% protein diet supplemented with *L. plantarum*; Data are presented as means ± SD (*n* = 6); a, b means indicates a significant difference at the *P* < 0.05 level.

The effects of dietary *L. plantarum* supplementation on serum antioxidant capacity of laying chicks are shown in Figure 3. Compared with the control group, the LP and LPL groups showed significantly higher SOD activity (*P* < 0.05). Compared with the control and LP groups, the LPL group had significantly increased GSH-Px and T-AOC activity (*P* < 0.05). There were no significant differences in POD, MDA, and CAT among the three groups (*P* > 0.05).

### Immune status

**Figure 4:**
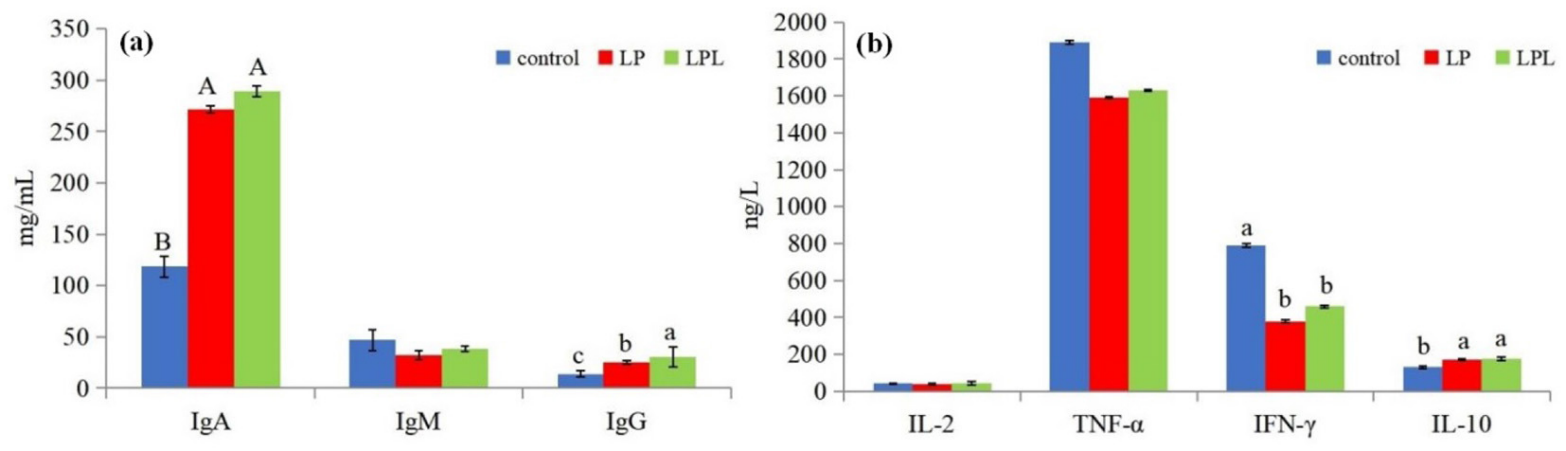
Effect of low protein diet containing *L. plantarum* on serum immune status of laying chicks. Control group was fed a basal diet containing 19% protein, LP group was fed a diet containing 17% protein, LPL group was fed with the 17% protein diet supplemented with *L. plantarum*; Data are presented as means ± SD (*n* = 6); a–c means indicates a significant difference at the *P* < 0.05 level and A, B means that at the *P* < 0.01 level.

The effects of dietary *L. plantarum* supplementation on serum immune status in laying chicks are shown in Figure 4. Compared with the control group, the LP and LPL groups showed significantly higher IgA and IgG content (*P* < 0.05), Compared with the LP group, IgG was significantly higher (*P* < 0.05) and for IgA, there was no significant differences (*P* > 0.05) in the LPL groups. There were no significant differences in IgM among the three groups (*P* > 0.05) (Figure 4A). Compared with the control group, the LP and LPL groups showed significantly higher IL-10 content and significantly lower IFN-γ content (*P* < 0.05). There were no significant differences in IL-2 and TNF-α among the three groups (*P* > 0.05) (Figure 4B).

### Gut microbiological analysis

**Figure 5:**
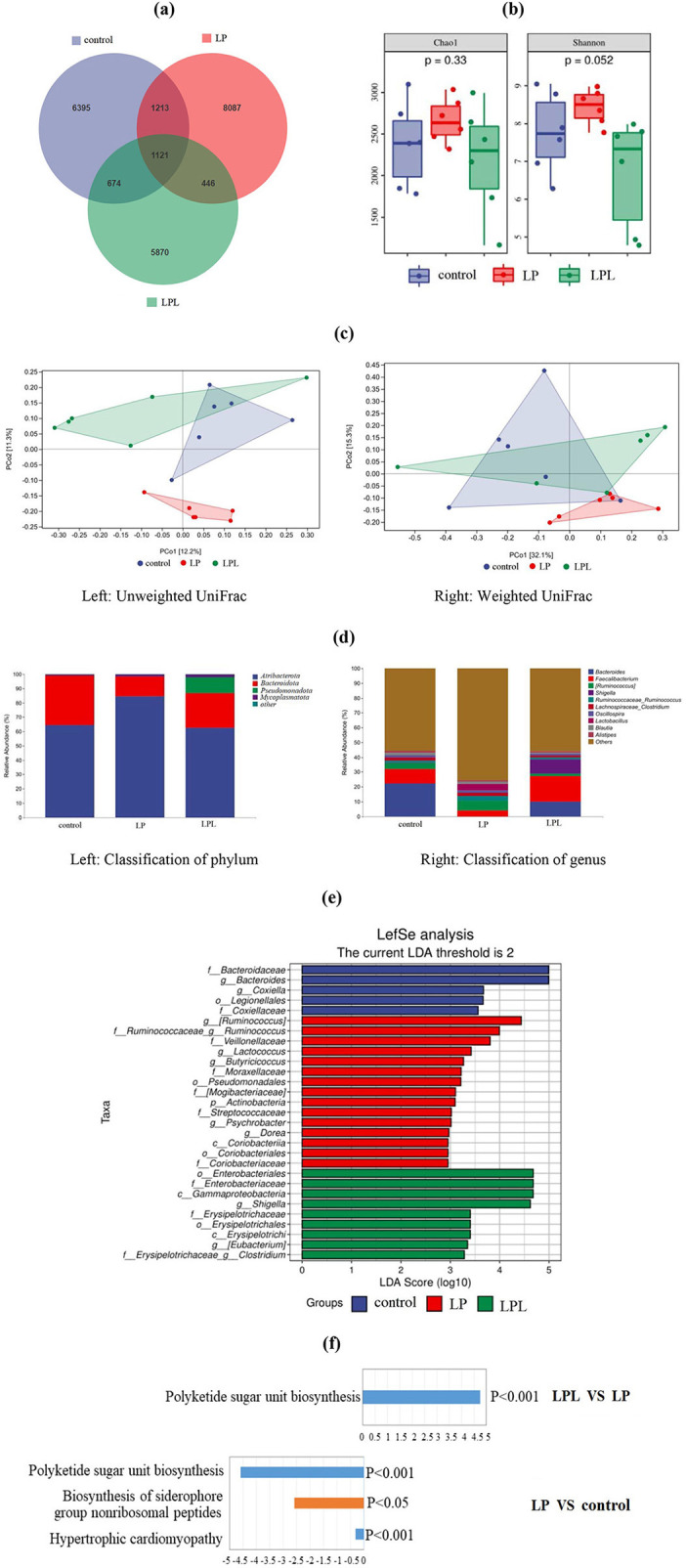
Effect of low-protein diet containing *L. plantarum* on cecal microbiota of laying chicks. Control group was fed a basal diet containing 19% protein, LP group was fed a diet containing 17% protein, LPL group was fed with the 17% protein diet supplemented with *L. plantarum*; Data are presented as means ± SD (*n* = 6). **(a)** Venn diagram displays the number of common and unique ASVs in the three groups of samples. **(b)** Bacterial alpha diversity based on Chao1 index and Shannon diversity. Chao1 represents species richness. Shannon diversity represents microbial community diversity. **(c)** Bray Curtis dissimilarity of cecal microbiota from the three groups were used for principal coordinate analysis (PCoA) plot (left: Unweighted UniFrac; right: Weighted UniFrac). Each dot represents the composition of the microbiota of each sample. Samples were grouped by colors as labels show. **(d)** Histogram displays the bacterial taxonomic composition at phylum (left) and genus (right) level. **(e)** LEfSe analysis of intestinal bacterial communities shows significantly different species in the three groups with LDA scores over 2. **(f)** Histogram of significant upregulation displays the metabolic pathway difference between the LPL and LP groups. Histogram of significantly down-regulation displays the metabolic pathway difference between LP and control groups.

A total of 2,060,624 input sequences were obtained, and 1,141,892 sequences were obtained after removing low-quality sequences, denoising, splicing, chimera removal and singleton removal, with an average length of 412 bp. A total of 23,806 ASV sequences were generated after 95% flattening, belonging to 12 phyla, 26 classes, 46 orders, 74 families, 136 genera, and 166 species. The results of intestinal microbiota diversity and metabolic pathway analysis are shown in Figure 5. The number of common and unique ASVs in the three groups of samples is shown in Figure 5A. There were 1,121 ASVs among the three groups, 6,395 ASVs in the control group, 8,087 ASVs in the 17% protein group, and 5,870 ASVs in the LPL group, respectively.

The alpha diversity analysis results of the three groups of samples are shown in Figure 5B. The Chao1 index is an index reflecting species richness, used to estimate the number of all species in a sample or environment. The higher the index, the higher the species richness. The Shannon index is an index reflecting the diversity of microfloar. The greater the index, the higher the diversity. The Chao1 index of the 17% protein group was higher than that of the control group, and the Chao1 index of the control group was higher than that of the LPL group, but there was no significant difference among all groups (*P* > 0.05). The Shannon index of the LP group was higher than that of the control group, and the Shannon index of the control group was higher than that of the LPL group, but there was no significant difference (*P* > 0.05). The Shannon index of the LP group was significantly higher than that of the LPL group (*P* < 0.05).

The beta diversity analysis results of the three groups are shown in Figure 5C. In the unweighted UniFrac distance analysis figure (left of Figure 5C), the dispersion between the LP group and the control group, and the LPL group, was large, and there was partial overlap between the control group and the LPL group. Weighted UniFrac distance analysis (right of Figure 5C) shows that there was some overlap between the control group and the LPL group, only a small overlap between the LP group and the control group, and no overlap between the LPL group and the control group.

In this study, the obtained ASVs were compared with the Greengenes database, and the bacteria with a relative abundance > 1% were selected to establish the species stacking bar chart. The species with a relative abundance < 1% in the species composition relationship were classified into the other group. The results of species classification based on the 1% phylum level are shown in Figure 5D left. In the control group, there were two phyla, the dominant phyla was *Atribacterota* (64.52%), followed by *Bacteroidota* (34.40%). There were three phyla in the LP group, among which *Atribacterota* (84.43%) were the dominant phyla, followed by *Bacteroidota* (13.36%) and *Mycoplasmatota* (1.10%). There were four phyla in the LPL group, among which *Atribacterota* (62.61%) were the dominant phyla, followed by *Bacteroidota* (24.07%), *Pseudomonadota* (11.27%), and *Mycoplasmatota* (1.66%). These phyla have been renamed according to the International Code of Prokaryotic Nomenclature (Oren and Garrity, 2021).

The results of the 1% genus level analysis for the three groups of samples are shown in Figure 5D right. In the control group, there were eight genera, in which the dominant genus was *Bacteroides* (22.30%), followed by *Faecalibacterium* (9.81%), *Lachnospireos-clostridium* (1.98%), *Oscillospira* (1.70%), *Blautia* (1.44%), *Ruminococceos-ruminococcus* (1.40%), *Ruminococcus* (1.14%), *Alistipes* (1.21%); There were eight genera in the LP group, among which the dominant genus was *Ruminococcus* (6.71%), followed by *Lactiplantibacillus* (4.47%), *Faecalibacterium* (3.82%), *Ruminococceos-ruminococcus* (3.17%), *Lachnospireos-clostridium* (2.00%), *Blautia* (1.67%), *Oscillospira* (1.59%), and *Alistipes* (1.01%). There were seven genera in the LPL group, among which the dominant genus was *Faecalibacterium* (17.29%), followed by *Bacteroides* (10.04%), *Shigella* (9.82%), *Ruminococcus* (1.58%), and *Bacteroides* (10.04%), *Oscillospira* (1.30%), *Lachnospireos-clostridium* (1.23%), and *Ruminococceos-ruminococcus* (1.16%).

The LEfSe analysis results are shown in Figure 5E. In the control group, the species with significant influence from high to low were *Bacteroidaceae, Bacteroides, Coxiella, Legionellales, and Coxillaceae*. In the LP group, the species with significant influence from high to low were *Ruminococcus, Ruminococcaceae- Ruminococcus, Veillonellaceae, Lactococcus, Butyricicoccus, Moraxellaceae, Pseudomonadales, Mogibacteriaceae, Actinobacteria, Streptococcaceae, Psychrobacter, Dorea, Coriobacteriia, Coriobacteriales, Coriobacteriaceae*. In the LPL group, the species with significant influence from high to low were *Enterobacteriales, Enterobacteriaceae, Gammaproteobacteria, Shigella, Erysipelotrichaceae, Erysipelotrichales, Erysipelotrichi, Eubacterium, Erysipelotrichaceae-Clostridium*.

In this experiment, PICRUSt2 analysis software was used to predict the 16sRNA gene sequence in the Kyoto Encyclopedia of Genes and Genomesdatabase (KEGG) and obtain the abundance data of metabolic pathways in these samples. The results of the metabolic pathway difference analysis are shown in Figure 5F. Compared with the control group, the bacterial community in the LP group had two significantly down-regulated metabolic pathways, namely hypertrophic cardiomyopathy (*P* < 0.001) and Polyketide sugar unit biosynthesis (*P* < 0.001), Biosynthesis of siderophore group nonribosomal peptides (*P* < 0.05) was a significantly down-regulated metabolic pathway. Compared with the LP group, the LPL group had a significantly upregulated metabolic pathway, namely polyketide sugar unit biosynthesis (*P* < 0.001). There were changes between the LPL and the control group in metabolic pathway, but not to a significant level.

## Discussion

In this study, we evaluated the effects of a low-protein diet supplemented with *L. plantarum* on growth performance, serum antioxidant capacity, immune factors, and intestinal flora of laying chicks (1–21 d). The following results were found: *L. plantarum* can improve the changes of ADG, ADFI, and F/G of laying chicks caused by a low-protein diet, improve liver index, maintain the growth performance of laying chicks, increase the activity of SOD, GSH-Px, the levels of IgA, IgG, and IL-10 in serum of laying chicks, and decrease the concentrations of IFN-γ, improve antioxidant capacity and cellular immunity. *L. plantarum* can improve the changes of intestinal flora abundance, diversity, and polyketose unit bioanabolic metabolism cause by a low-protein diet, and improve the intestinal flora balance.

### Effects of dietary composition on growth performance of laying chicks

Dietary protein level is an important factor to determine the growth and development of young animals. Providing a good protein level for animals can improve production performance and disease resistance (Freitas et al., 2023; Xiao et al., 2023). The ADG, ADFI, and F/G are important indices to evaluate the growth performance of poultry, which is the first step to evaluate the successful application of a low-protein diet. De Cesare et al. (2019) showed that a 7% reduction in dietary crude protein levels in broilers aged 0–20 days had no effect on performance but increased feed conversion. Kalavathy et al. (2003) found that diets supplemented with a 0.1% mixture of chicken derived lactic acid bacteria could significantly reduce the ratio of feed to gain of broilers in the early period (1–21 days of age), and significantly increase the ADG of broilers in the early period. Song et al. (2022) showed that adding *L. plantarum* had no significant effect on the growth performance of 1-day-old broilers. The results showed that LP group and LPL group did not affect the growth performance of laying hens (1–21 d), mainly digestive and immune system development (Ayalew et al., 2023; Liu Y. et al., 2023). Although LP and LPL reduce protein content, *L. plantarum* increases feed availability and balances protein supply and demand, meeting the protein and energy requirements of the early-stage laying hens, so that their growth performance is not affected.

### Effects of dietary composition on organ index of laying chicks

Heart can maintain the circulation of the blood, liver is the metabolism site of the three major nutrients important digestive, spleen and bursa of fabricius is poultry important peripheral immune organs and central immune organ, participate in the cellular immunity and humoral immunity, determining the level of poultry immune directly. The organ index is mainly related to animal species and dietary nutrition level. The size of the organ index directly reflects the growth and development of visceral organs and the strength of organ function, and then reflects the health status of the animal's body (Alabi et al., 2023). Wang et al. (2013) studied 1 d broilers of different breeds and different protein levels and found that the organ indices of the heart, liver, spleen, lung, kidney, and bursa of fabricius were associated with the breeds, but not with a low-protein diet. Wang J. et al. (2023) showed that the bursal index and spleen index were significantly increased in yellow-finned broilers aged 1–21 d by supplementing the diet with *L. plantarum*. The experiment found that *L. plantarum* could improve the liver index of the LP group, which may be related to the improvement of feed efficiency by *L. plantarum*, promoting liver development, nutrition, and metabolism.

### Effects of dietary components on antioxidant capacity of laying chicks

Under stress, the bodies of livestock and poultry will produce reactive oxygen species and free radicals that will cause oxidative damage to them. MDA is an important index reflecting the degree of oxidative damage of fat. Antioxidant enzymes are important biological active components that protect tissues from peroxidation loss, and they are the material basis of the antioxidant ability of the body. Oxidative damage can be alleviated by antioxidant enzymes such as SOD, POD, T-AOC, GSH-Px, and CAT (Liu X. et al., 2023). Sharifi et al. (2016) showed that broilers fed a low-protein diet (30 g/kg reduction in crude protein) at high altitudes for 42 days produced higher levels of ROS in the serum than those fed a normal protein diet, the level of serum MDA increased significantly, which aggravated the oxidative damage. Shen et al. (2014) showed that *Lactobacillus* can increase glutathione peroxidase activity in the serum and liver of 21 d broilers and decrease MDA content in the serum of 42 d broilers to promote antioxidant capacity. Deraz et al. (2019) also found that supplementation with *L. plantarum* in broiler diets resulted in increased T-AOC concentrations in serum and decreased MDA. Consistent with the above results, this experiment shows that both a low-protein diet and *Lactiplantibacillus* could improve the antioxidant capacity of laying chicks (1–21 d), the activities of SOD, T-AOC, and GSH-Px were increased significantly, the effect of *L. plantarum* was more significant, and the changes of oxidative metabolism *in vivo* may be related to the decrease of amino acids in the low-protein diet and the difference of peroxide scavenging ability of different *Lactiplantibacillus* species.

### Effects of dietary components on immune factors of laying chicks

Immunoglobulins (IgM, IgG, and IgA) as the main functional products of the animal immune response, have a wide range of responses to foreign antigens the content being more significant for the change of immune level. IL-2 is an immune cytokine that promotes the growth and proliferation of T lymphocytes, B lymphocytes, and the production of antibodies, IL-10 acts as an immunosuppressant by inducing the differentiation of cytotoxic T lymphocytes, NK cells, and other killer cells and by stimulating the body to secrete IFN-γ, TNF-α, etc. a variety of cytokines that inhibit the production of monocytes. Yang et al. (2024) and Wang et al. (2018) found that the addition of *Lactobacillus* to diets increased serum immunoglobulin concentrations in broilers to increase humoral immunity. Wang et al. (Wang et al., 2018) showed that adding *L. plantarum* to the diet can improve immune function by increasing serum IgG and IgA levels in yellow-feather broilers. Sigolo et al. (2017) showed that a 2.5% reduction in dietary crude protein content compared with the recommended value did not adversely affect the immune capacity of 1–42 d broilers. Chen et al. (2012) found that feeding a *Lactobacillus*-containing diet to salmonella-infected chickens reduced the expression levels of IL-1β, IL-6, and IFN-γ and increased the expression levels of IL-10, consistent with the results in this trial. Compared with the control group, the low-protein diet and *Lactobacillus* could increase the level of serum antibodies (IgA and IgG) and the anti-inflammatory factor (IL-10) content, but the pro-inflammatory factor (IFN-γ) content was decreased, which resulted in the body's being in a higher immune state thus improving disease resistance. This may be due to changes in the composition of the diet, making the immune system more sensitive, and the body's anti-inflammatory and pro-inflammatory factors' dynamic equilibrium, the exact reason for which remains to be explored further.

### Effects of dietary components on cecal microbiota of laying chicks

Gastrointestinal microorganisms and their metabolites play an important role in enhancing nutrient absorption and the immune system in livestock and poultry. Previous experiments have shown that there is a close relationship between animal performance and dietary composition, intestinal microbiota and host metabolic changes (Yin et al., 2023). The microbial communities of broiler chickens are different from those of laying hens. The composition of the gut microbiota varies with age, genotype and production system (Ducatelle et al., 2023; England et al., 2024). Dong et al. (2017) studied dietary protein levels in 28-week-old laying hens and found that a reduction in dietary protein levels from 16 to 14% reduced gut bacterial diversity (*P* < 0.05). The alpha diversity and beta diversity of intestinal microflora in three groups of laying chicks fed different diets were compared by 16S amplicon sequencing, and it was found that chicks of the LP group had the highest abundance and diversity of intestinal flora, which was not consistent with the results of Dong et al. (2017). This may be due to the different age of the layers and the different protein content in the diet, as the structure of gastrointestinal flora is unstable and the utilization of protein is different in layer chickens compared with adult layers (Choi and Kim, 2023). LP is more suitable for the nutritional requirement of gastrointestinal flora in layer chickens, good for intestinal health. The abundance and diversity of intestinal microflora in the LPL group were similar to those in the control group, which may be due to the increased proportion of *L. plantarum* in the intestinal tract and the predominance of *L. plantarum*. Competitive rejection inhibits the proliferation of some harmful bacteria in the gut and provides a good environment for the growth of beneficial bacteria (Dixon et al., 2022), resulting in a decrease in both the abundance and diversity of the intestinal flora.

*Atribacterota* and *Bacteroidota* are the most important bacterial components in the gut, and the ratio of the two can usually reflect the state of microbial homeostasis (Wang X. Y. et al., 2023). Rist et al. (2014) showed that reducing dietary protein levels significantly increased the abundance of *Enterobacteriaceae* and *Bacteroides* in ileal digesta of weaned piglets, and decreased the abundance of *Enterobacteriaceae* and *Bacteroides* in fecal samples. Yu et al. (2019) fed weaned piglets a low-protein diet supplemented with lysine, methionine, threonine, and tryptophan and found that jejunal and colonic microbiota were not affected at the phylum or genus level in any diet. The results showed that a low-protein diet could increase the relative abundance of *Atribacterota* and decrease the relative abundance of *Bacteroidota* in cecal microbiota of chicks. *L. plantarum* can reduce the effect of low-protein diet on *Atribacterota* and increase the relative abundance of *Pseudomonadota* in cecal microbiota of egg chicks, which is consistent with the results of Zhang et al. (2019), and Song et al. (2022) The relative abundance of *Pseudomonadota* was 11.27% in the cecal flora of the LPL group, and *Pseudomonadota* mostly played an active role in the digestion and utilization of starch in the host and energy production in the body (Chen et al., 2023). These inconsistencies may be related to diet composition, animal species and microbial colonization of different intestinal segments.

The LEfSe map shows the species that were significantly enriched within each group and their degree of importance. The results showed that there were significant differences among the three groups, which may be related to the effect of a low-protein diet containing *L. plantarum* on the intestinal microflora of laying chickens, consistent with the study by Rehman et al. (2007) PICRUSt was used to study functional differences in the microbiota to assess metabolic alterations induced by low-protein diets containing *L. plantarum*. Three significantly down-regulated metabolic pathways including hypertrophic cardiomyopathy, polyketide sugar unit biosynthesis and biosynthesis of siderophore group were found in the LP group. The significantly upregulated metabolic pathway of polyketide sugar unit biosynthesis was found in LPL group. This may be related to the changes in intestinal microbiota structure by LP, and the absorption of amino acids and other nutrients by *L. plantarum* through other metabolic pathways, thus improving the feed utilization rate of chickens, which is consistent with the results of Yan et al. (2017).

## Conclusion

In conclusion, the reduction of protein content will result in significant differences in the richness and diversity of intestinal flora, resulting in changes in community structure and metabolic pathway of intestinal bacteria. *L. plantarum* can maintain the balance of intestinal flora, up-regulate the metabolic pathway of polyketide sugar unit biosynthesis, reduce the metabolic disorder caused by low protein, and keep the intestinal tract healthy. Therefore, the low-protein (17%) diet with 1.0 × 10^9^ CFU/kg *L. plantarum* (LPL) can meet nutritional need by regulating the oxidation resistance and balance of intestinal flora in chickens. It is feasible that *L. plantarum* can be used as feed additive in the low protein diet.

## Data Availability

The original contributions presented in the study are publicly available. This data can be found here: https://www.ncbi.nlm.nih.gov/sra, BioProject accession number: PRJNA1215420; SRP number: SRP560108.
